# Contrasting Patterns of Coral Bleaching Susceptibility in 2010 Suggest an Adaptive Response to Thermal Stress

**DOI:** 10.1371/journal.pone.0033353

**Published:** 2012-03-09

**Authors:** James R. Guest, Andrew H. Baird, Jeffrey A. Maynard, Efin Muttaqin, Alasdair J. Edwards, Stuart J. Campbell, Katie Yewdall, Yang Amri Affendi, Loke Ming Chou

**Affiliations:** 1 Marine Biology Laboratory, Department of Biological Sciences, National University of Singapore, Singapore, Singapore; 2 Australian Research Council Centre of Excellence for Coral Reef Studies, James Cook University of North Queensland, Townsville, Australia; 3 Centre National de la Recherche Scientifique, Centre de Recherches Insulaires et Observatoire de l'Environnement, Moorea, French Polynesia; 4 The Wildlife Conservation Society, Indonesian Marine Program, Bogor, Indonesia; 5 School of Biology, Newcastle University, Newcastle-upon-Tyne, United Kingdom; 6 Blue Ventures, Aberdeen Centre, London, United Kingdom; 7 Institute of Biological Sciences, Faculty of Science, Universiti Malaya, Kuala Lumpur, Malaysia; University of Texas, United States of America

## Abstract

**Background:**

Coral bleaching events vary in severity, however, to date, the hierarchy of susceptibility to bleaching among coral taxa has been consistent over a broad geographic range and among bleaching episodes. Here we examine the extent of spatial and temporal variation in thermal tolerance among scleractinian coral taxa and between locations during the 2010 thermally induced, large-scale bleaching event in South East Asia.

**Methodology/Principal Findings:**

Surveys to estimate the bleaching and mortality indices of coral genera were carried out at three locations with contrasting thermal and bleaching histories. Despite the magnitude of thermal stress being similar among locations in 2010, there was a remarkable contrast in the patterns of bleaching susceptibility. Comparisons of bleaching susceptibility within coral taxa and among locations revealed no significant differences between locations with similar thermal histories, but significant differences between locations with contrasting thermal histories (Friedman = 34.97; p<0.001). Bleaching was much less severe at locations that bleached during 1998, that had greater historical temperature variability and lower rates of warming. Remarkably, *Acropora* and *Pocillopora*, taxa that are typically highly susceptible, although among the most susceptible in Pulau Weh (Sumatra, Indonesia) where respectively, 94% and 87% of colonies died, were among the least susceptible in Singapore, where only 5% and 12% of colonies died.

**Conclusions/Significance:**

The pattern of susceptibility among coral genera documented here is unprecedented. A parsimonious explanation for these results is that coral populations that bleached during the last major warming event in 1998 have adapted and/or acclimatised to thermal stress. These data also lend support to the hypothesis that corals in regions subject to more variable temperature regimes are more resistant to thermal stress than those in less variable environments.

## Introduction

Coral reefs are critically important for the ecosystem goods and services they provide to maritime tropical and subtropical nations [1]. However, major coral bleaching events – caused by a breakdown in the relationship between scleractinian corals and their algal symbionts – have led to widespread coral mortality on reefs in recent decades [2]. Global warming poses a particularly significant threat to the future of coral reef ecosystems because large-scale coral bleaching episodes are strongly correlated with elevated sea temperatures [3], [4]. Indeed, among Earth's ecosystems, coral reefs are one of the most severely threatened by global warming [5].

Coral bleaching severity varies in space and time as a consequence of the magnitude of thermal stress [6], levels of irradiance [7], [8], symbiont types [9], the species composition of the coral assemblage [10], [11], [12] and thermal history of the site [13], [14]. Species composition is one of the strongest drivers of this variation due to a predictable hierarchy of susceptibility among coral taxa [10], [11], [12]. Fast growing branching taxa, such as *Acropora* and *Pocillopora*, are normally highly susceptible to thermal stress; they bleach rapidly and experience high rates of whole colony mortality [15]. In contrast, massive taxa such as *Porites* and some faviids are more resistant to bleaching, they take longer to bleach, and although they may stay bleached for longer, few entire colonies die [15]. This consistency has led to the prediction that hardier, slow-growing massive species will replace less hardy, fast-growing branching species on reefs in the future [10], [16]. The thermal history of a site may also play an important role in determining bleaching severity. For example, on reefs with naturally higher temperature fluctuations, corals are frequently exposed to stressful temperatures for short periods, and this may lead to greater tolerance during episodes of more prolonged thermal stress [14], [17].

Scleractinian corals are the major framework builders of reefs and provide most of the structural complexity in reef ecosystems. Therefore, the capacity of coral species to adapt and acclimatise to increasing episodes of thermal stress will greatly influence rates of reef degradation [5]. Several studies cite repeated bleaching episodes in the same coral assemblages, the increasing scale and frequency of coral bleaching and the low overall evolutionary potential of scleractinians as evidence that corals have exhausted their capacity to adapt to rising sea temperatures [18], [19]. In contrast, other studies show considerable spatial and temporal variation in bleaching susceptibility within scleractinian taxa, suggesting an underappreciated capacity for corals to adapt and/or acclimatise to thermal stress [20].

If the hypothesis that corals still have the capacity to adapt to elevated sea temperatures is correct, we would expect to find increases in thermal tolerance on reefs that have previously experienced major bleaching with the most susceptible species exhibiting the greatest increases in thermal tolerance [21]. Furthermore, we would expect reefs in more thermally variable environments to bleach less severely during episodes of elevated sea temperatures [14]. Here we examine the bleaching and mortality responses of corals at sites with contrasting thermal histories during a large-scale bleaching event in 2010. Our data provide evidence in support of both hypotheses as we documented an unprecedented reversal in the susceptibility of coral genera, but only at sites where bleaching occurred in 1998. Furthermore we show that corals generally bleached less severely at locations where temperature variability has been greater and warming rates lower over the last 60 years.

## Results

### Coral bleaching and mortality response

**Figure 1:**
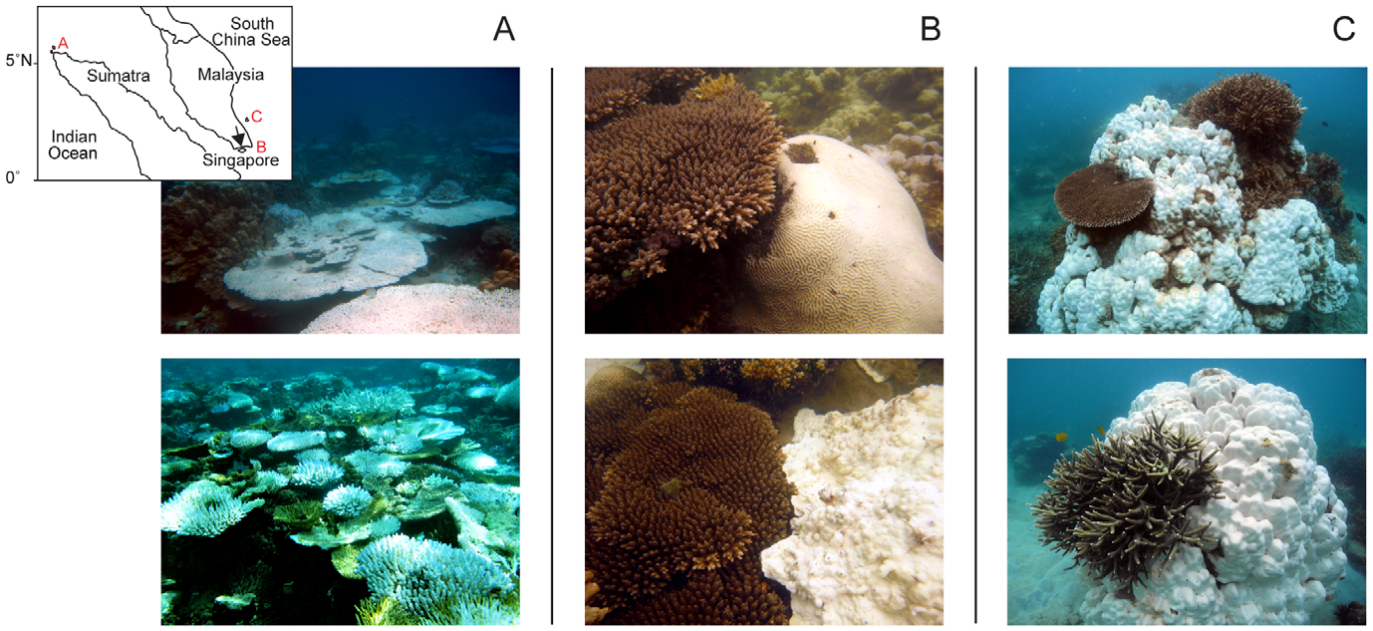
Contrasting coral bleaching patterns during 2010. Extensive stands of bleached *Acropora* colonies from (A) Pulau Weh, north Sumatra, Indonesia where patterns in bleaching susceptibility were normal. Reversals in bleaching susceptibility gradients were observed in (B) Singapore and (C) Tioman Island, Malaysia, where healthy *Acropora* colonies were found adjacent to bleached encrusting, foliose and massive colonies: corals which are usually relatively resistant to bleaching. The inset map shows the three study locations.

A major thermal anomaly that began in May 2010 led to extensive coral bleaching on reefs at sites in South East Asia (http://coralreefwatch.noaa.gov/satellite/index.html). In Pulau Weh, north Sumatra, in May and June 2010 we observed patterns of susceptibility that were similar to the last major bleaching episode in the Indo-West Pacific in 1998, with severe bleaching and mortality of *Acropora* and *Pocillopora* (Figure 1A). However, at sites in Malaysia and Singapore in July 2010 we found these taxa were surprisingly, largely unaffected by bleaching whereas massive taxa bleached severely, as expected given the level of thermal stress (Figure 1B, C).

**Figure 2:**
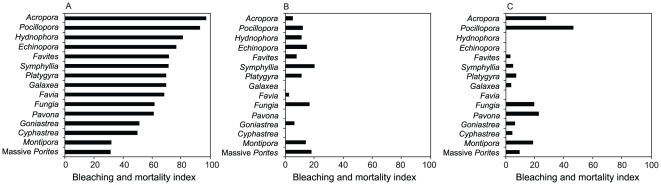
Comparison of bleaching and mortality indices among locations during 2010. Graphs compare the bleaching and mortality indices (BMI) of 15 coral genera that had >5 colonies recorded during surveys at each location for (A) Pulau Weh, (B) Singapore and (C) Tioman Island in 2010. Data used to estimate BMI are from Table 2.

Analysis of the bleaching and mortality response (BMI) of coral genera [12] (and see methods) carried out at the three locations in 2010 and the Great Barrier Reef (GBR) in 1998 revealed a significant difference in bleaching susceptibility among locations (Friedman test = 34.97, p<0.0001). Dunn's post-hoc multiple comparisons revealed that there were no significant differences between Pulau Weh in 2010 and the GBR in 1998 [11] (Table 1). Similarly, there were no significant differences between the two South China Sea locations in 2010 (Table 1). However, bleaching susceptibilities of coral taxa at both Singapore and Tioman Island were significantly different from Pulau Weh (p<0.001, Table 1). The coral taxa *Acropora* and *Pocillopora* were the most susceptible in Pulau Weh but among the least susceptible in Singapore. In Pulau Weh, 94% of *Acropora* and 87% of *Pocillopora* colonies were recorded as recently dead, compared to only 5% of *Acropora* and 12% of *Pocillopora* in Singapore (Table 2, Figure 2). In Tioman Island, *Acropora* and *Pocillopora* were much less affected than at Pulau Weh, with 28% and 36% respectively of colonies recently dead, however these taxa still ranked among the most susceptible at this location (Table 2, Figure 2).

**Table 1:** Results of Friedman test and Dunn's multiple comparisons of bleaching and mortality response within taxa and among locations.

Dunn's multiple comparisons	Difference in rank sums	*P*
Pulau Weh 2010 vs. GBR 1998	13	ns
Pulau Weh vs. Tioman Island	34.5	***
Pulau Weh vs. Singapore	34.5	***
Tioman Island vs. Singapore	0	ns

Friedman statistic = 34.97, n = 15, p<0.0001.

*** = p<0.001, GBR = Great Barrier Reef.

**Table 2:** Bleaching and mortality response of coral genera from three study locations.

		Pulau Weh						Singapore						Tioman Island				
Genus	Normal(%)	Moderate(%)	Severe(%)	Dead(%)	*n*	BMI	Normal(%)	Moderate(%)	Severe(%)	Dead(%)	*n*	BMI	Normal(%)	Moderate(%)	Severe(%)	Dead(%)	*n*	BMI
*Acropora*	0	2	4	94	*2696*	**97**	95	1	0	5	*448*	**5**	70	3	1	26	*632*	**28**
*Pocillopora*	0	7	6	87	*689*	**93**	79	13	0	7	*68*	**12**	39	21	2	38	*87*	**46**
*Hydnophora*	3	3	41	53	*32*	**81**	78	17	0	6	*18*	**11**	100	0	0	0	*8*	**0**
*Echinopora*	0	27	17	57	*30*	**77**	56	44	0	0	*18*	**15**	100	0	0	0	*8*	**0**
*Favites*	1	4	73	21	*406*	**71**	86	8	4	3	*80*	**8**	91	9	0	0	*53*	**3**
*Symphyllia*	0	0	86	14	*22*	**71**	66	18	6	10	*50*	**20**	86	14	0	0	*7*	**5**
*Platygyra*	0	5	80	15	*55*	**70**	73	22	6	0	*88*	**11**	86	9	2	2	*43*	**7**
*Galaxea*	5	5	65	25	*77*	**70**	100	0	0	0	*77*	**0**	91	7	2	0	*56*	**4**
*Favia*	1	8	76	15	*264*	**68**	93	7	0	0	*44*	**2**	100	0	0	0	*19*	**0**
*Porites* (branching)	15	25	0	60	*291*	**68**	50	0	0	50	*2*	**50**	54	0	0	46	*28*	**46**
*Fungia*	0	30	56	15	*27*	**62**	75	11	3	11	*36*	**17**	54	38	5	4	*128*	**20**
*Pavona*	17	0	67	17	*6*	**61**	100	0	0	0	*18*	**0**	66	11	11	11	*71*	**23**
*Acanthastrea*	5	20	64	11	*103*	**60**	90	5	5	0	*21*	**5**	67	33	0	0	*3*	**11**
*Goniastrea*	15	23	54	8	*13*	**51**	85	12	3	0	*66*	**6**	90	6	0	4	*71*	**6**
*Cyphastrea*	0	75	0	25	*8*	**50**	100	0	0	0	*9*	**0**	87	13	0	0	*15*	**4**
*Goniopora*	14	36	43	7	*42*	**48**	86	9	5	0	*22*	**6**	100	0	0	0	*1*	**0**
*Montastraea*	19	40	37	3	*62*	**41**	50	50	0	0	*4*	**17**	90	10	0	0	*21*	**3**
*Montipora*	43	37	1	19	*589*	**32**	81	7	1	11	*134*	**14**	78	4	0	17	*418*	**19**
*Porites* (massive)	30	53	10	7	*3246*	**31**	65	19	13	3	*69*	**18**	74	23	2	0	*86*	**9**
*Diploastrea*	20	70	10	0	*20*	**30**	100	0	0	0	*13*	**0**	100	0	0	0	*3*	**0**

Data are proportions of colonies (%) that were not bleached (normal), moderately bleached (uniformly pale or <50% of colony bleached), severely bleached (>50% of colony bleached) or recently dead and the bleaching and mortality index (BMI) for corals in all genera that were surveyed in Pulau Weh (21 weeks after temperatures exceeded maximum monthly mean), Singapore and Tioman Island (25 weeks after temperatures exceeded maximum monthly mean). BMI for Pulau Weh is ranked in descending order for comparison within taxa and among sites.

In addition to the reversal in the hierarchy of susceptibility among taxa between Singapore and Pulau Weh, most genera in Pulau Weh in 2010 were more severely affected than at the other two sites. In Pulau Weh, 44.7±5.04%, (mean ± SE) of colonies were recently dead during surveys whereas in Singapore and Tioman Island only 4.2±0.71% and 15.4±2.47% (mean ± SE) of colonies were recently dead (Table 2). Several taxa in Pulau Weh had a high proportion of dead colonies (i.e. >50% of colonies recently dead) relative to the other locations. For example, in Pulau Weh, 53% of *Hydnophora* and 57% of *Echinopora* colonies were recently dead, whereas in Singapore only 6% of *Hydnophora* colonies were dead and in Tioman Island no colonies of either genera were found dead (Table 2). The massive faviid genera *Cyphastrea*, *Favia* and *Platygyra*, although less severely affected than other taxa, were also more severely affected in Pulau Weh compared to Singapore and Tioman Island with 15% to 25% of colonies recently dead in Pulau Weh compared to <2% of colonies in the South China Sea sites (Table 2). Conversely branching *Porites* were severely affected in all locations with the proportion of recently dead colonies being 60% in Pulau Weh, 50% in Singapore and 46% in Tioman Island; whereas four massive coral taxa (*Diploastrea*, massive *Porites*, *Montastraea* and *Goniastrea*) had similar, but less severe, responses at all three locations with between 0% and 8% of colonies recently dead (Table 2).

### Short-term thermal history of study sites

**Figure 3:**
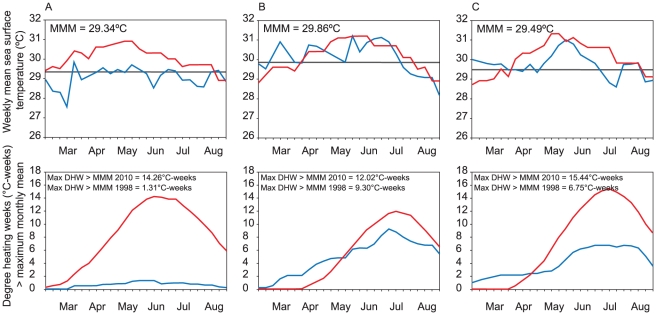
Comparison of sea temperatures and thermal stress during 1998 and 2010. Weekly mean sea surface temperatures (°C) and maximum monthly mean (MMM) temperatures (top row) and thermal stress in degree heating weeks (DHW) (°C-weeks) above MMM in 1998 and 2010 (bottom row) for (A) Pulau Weh; (B) Singapore; and (C) Tioman Island. Blue lines are SST and DHW from 1998, red lines are from 2010 and the gray line is MMM. Values for maximum degree DHW > MMM in 1998 and 2010 are shown.

Coral bleaching occurs when sea surface temperatures (SST) exceed climatological maximum monthly mean (MMM) for prolonged periods and the extent of thermal stress is typically expressed in terms of degree heating weeks (DHW) [22]. Remotely sensed data from Pulau Weh, (the location most severely affected by bleaching in 2010), showed that SST and DHW rarely exceeded the MMM in 1998 (Figure 3A), whereas in both Singapore and Tioman Island they did for prolonged periods (Figure 3B, C). Total DHW above MMM were 5 and 7 times higher in Tioman Island and Singapore than in Pulau Weh in 1998. In contrast, thermal stress was high and similar at all locations in 2010, with maximum DHW above MMM ranging from 12.02 to 15.44°C-weeks (Figure 3).

### Long-term thermal history of study sites

**Figure 4:**
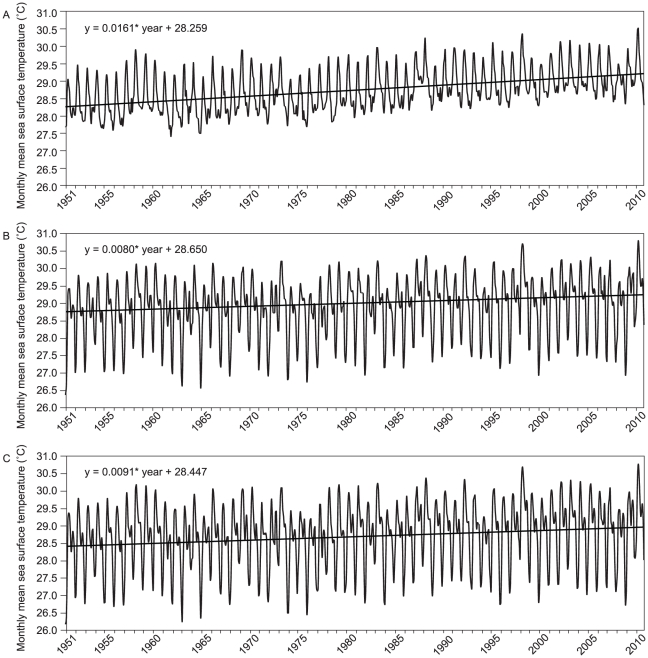
Comparison of long-term thermal histories. Monthly mean sea surface temperatures from January 1951 to December 2010 for (A) Pulau Weh; (B) Singapore and (C) Tioman Island. The linear regression is shown by the straight black line and the equation shows the average rate of temperature increase.

Long-term thermal histories for each location, determined by examining monthly mean SST data for the 1° longitude–latitude squares that encompassed each study location, indicate that despite similar mean temperatures in Singapore (mean 28.89 ± SD 0.83°C), Tioman Island (mean 28.72 ± SD 0.90°C) and Pulau Weh (mean 28.74 ± SD 0.59°C), only the thermal histories of the two South China Sea locations are similar (Figure 4). For example, annual variability (standard deviation) at the South China Sea locations is 41–52% higher than that of Pulau Weh. Furthermore, the decadal rates of warming over the last 60 years for Singapore (0.08°C decade^−1^) and Tioman Island (0.09°C decade^−1^) are close to half the rate for Pulau Weh (0.16°C decade^−1^).

## Discussion

Fast growing branching coral taxa, such as *Acropora* and *Pocillopora*, are normally highly susceptible to thermal stress and to date there has been a predictable hierarchy of bleaching susceptibility that was consistent over a wide geographic range and among bleaching events [10], [11], [12]. The hierarchy of susceptibility in Pulau Weh in 2010 was typical of previous bleaching episodes, for example the 1998 event on the GBR [11], but was in marked contrast with the patterns of susceptibility observed in Singapore and Tioman Island (see Figure 1). Comparisons of the BMI of taxa among locations revealed significantly different patterns between Pulau Weh and the South China Sea locations and confirmed a reversal in the normal patterns of susceptibility between Singapore and Pulau Weh. This is the first time such a reversal has been reported during a major warming-induced bleaching event. The remotely sensed temperature data corroborate reports indicating that corals in Singapore and Tioman Island bleached in 1998 but those in Pulau Weh did not. Extensive bleaching was documented in Singapore, in several nearby Indonesian sites [23] and Tioman Island [24] during 1998; whereas there are no reports of bleaching from Pulau Weh prior to 2010 despite numerous reports from elsewhere in the Indonesian archipelago (www.reefbase.org). Local dive operators have not witnessed mass bleaching in the area in the last 30 years and in nearby Andaman Sea sites, severe bleaching was not observed during 1997–1998 [8]. A parsimonious explanation for the contrasting bleaching responses among locations, therefore, is that removal of susceptible individuals from populations that bleached during 1998 in Singapore and Tioman Island, followed by reproduction and successful recruitment of the remaining, more thermally tolerant individuals, has led to adaptation through natural selection within an ecological time frame [13], [25].

Recurring bleaching episodes of increasing magnitude and frequency within coral assemblages have been cited as evidence that corals have exhausted their capacity to adapt and it is often stated that the generation times of corals are too long to allow rapid adaptation to a changing climate [18], [19]. In contrast, a growing body of evidence indicates that the capacity for adaptation and acclimatisation in corals has been underestimated [13], [21], [26]. Even for highly susceptible coral species, variation in specific characteristics of the symbiotic zooxanthellae [27] and the coral host [28] lead to different bleaching responses among colonies. Selective mortality among individuals within populations suggests there is sufficient genetic variability upon which natural selection can act [29]. Several studies have documented increasing thermal tolerance and declining rates of bleaching induced mortality over successive bleaching episodes [21], [30]. Similarly, thermal history and previous exposure to thermal stress have been shown to determine bleaching responses to contemporary thermal stress [13]. The most compelling evidence of an adaptive response at our study locations is that the taxa that showed the greatest contrast in response (*Acropora* and *Pocillopora*), have life history traits most likely to lead to rapid adaptation. For example, these taxa become sexually mature within 2 to 3 years [31], [32] and typically experience high rates of whole colony mortality following thermal stress [15].

Most taxa bleached much less severely and far fewer corals died in Singapore and at Tioman Island than in Pulau Weh in 2010. The 2010 episode in Pulau Weh was greater in magnitude compared to previous major bleaching episodes, such as that on the GBR in 1998 [11], and surveys from other sites in the Andaman Sea indicate that the 2010 event is the most severe for this region on record [8], [33]. The differences in overall bleaching severity among the three study locations in 2010 are not readily explained by differences in the magnitude of the thermal anomaly but may have been influenced by long-term differences in thermal histories at each location. In environments with naturally higher temperature fluctuations, the coral holobiont is frequently exposed to stressful temperatures for short durations, and this may lead to greater tolerance during episodes of prolonged thermal stress [14], [17], [34]. Consequently, acclimatisation of corals driven by greater thermal variability and facilitated by slower warming rates may also have led to overall differences in the severity of bleaching responses among locations. If our findings apply more generally then locations that are more resistant to bleaching can be identified from their thermal histories. Such knowledge can be used to inform protected area planning by aiding in the identification of sites with lower relative vulnerability to global warming [35], [36].

It is often stated that corals have exhausted their capacity to adapt to thermal stress [18], [19]. Here we provide evidence in support of the alternative hypothesis, i.e., taxa that, to date, have been consistently the most thermally susceptible possess an underappreciated capacity for adaptation to thermal stress [26]. Identification of genes that respond to thermal stress and are under selection, followed by studies to quantify changes in gene expression and gene frequency among coral populations are required to assess the likelihood that adaptation has driven the response seen in these populations [37]. Our study also highlights the critical importance of comparing rates of bleaching induced mortality within coral populations and spanning repeated bleaching episodes; indeed, such data are essential if we hope to assess the capacity of coral populations to adapt to rising temperatures. We cannot rule out the possibility that differences in irradiance [8], turbidity and thermal stress among locations also contributed to the spatial variation in the severity of bleaching in 2010. For example lower bleaching severity in Singapore may in part be explained by lower thermal stress and higher turbidity relative to the other sites – however, the differences in environmental stress do not explain the reversal in the hierarchy of susceptibility among taxa.

An adaptive response in certain taxa at a few locations does not mean that the global threat to reefs from climate change has lessened. There are likely to be limits to thermal adaptation and acclimatisation, and these may incur costs in life history traits such as growth, fecundity and competitive ability [20]. In addition, reefs continue to be threatened by numerous other factors including overfishing, pollution, disease, acidification, and severe storms [16]. The results of the present study do indicate however that the effects of bleaching will not be as uniform as anticipated [20] and fast-growing branching taxa such as *Acropora* and *Pocillopora* are likely to persist in some locations despite increases in the frequency of thermal stress events.

## Materials and Methods

### Coral bleaching and mortality response surveys

Surveys to assess the bleaching and mortality response were carried out on reefs at three locations: around Pulau Weh, northwest Sumatra, Indonesia (5°50′N, 95°20′E); southeast of Singapore (1°10′N, 103°50E); and at Tioman Island, off the east coast of Peninsular Malaysia (2°49′N, 104°08′E) (Figure 1). The marked contrast in hierarchy of taxa susceptibility was first noted during visits to the three locations during May, June and July 2010 (Figure 1). Subsequently, surveys of the bleaching and mortality responses (BMI) [12] of corals were carried out at 13 sites in Pulau Weh between 26 and 31 July 2010, four sites southeast of Singapore between 4 and 13 October 2010 and five sites at Tioman Island between 9 and 11 of October 2010. In relation to the time of the onset of the thermal anomaly, surveys were +21 weeks (Pulau Weh) and +25 weeks (Singapore and Tioman Island) after sea temperatures exceeded the climatological MMM. Depth at the survey sites ranged from 1 to 6 m. Two-metre radius survey plots were selected by swimming in a haphazardly chosen direction and for a random number of fin-kicks between 3 and 20. All colonies within the survey plot were included and this process was repeated for between 40 min and 2 h at each site. Each colony within the survey plot was identified to genus and bleaching status [11] was recorded as follows: 1, healthy = no bleaching; 2, moderately bleached = colony pale or less than 50% of surface area bleached; 3, severe = colony greater than 50% bleached; and 4, recently dead. An index of the susceptibility to the bleaching event for each taxon and location was calculated [12]. A bleaching and mortality index (BMI) based on the four coral status categories described above and was calculated as follows:
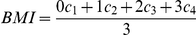
where *c*_1_ to *c*_4_ are the four coral status categories expressed as the proportion of colonies (%) surveyed arranged in order from normal (unbleached) to recently dead. The sum of the four categories is divided by 3 to produce an index that is on a scale from 0 to 100 [12]. A non-parametric Friedman test, followed by Dunn's post-hoc multiple comparison test was carried out to compare BMI within taxa and among the three study locations. In addition, to compare the bleaching and mortality response between Pulau Weh in 2010 and previous major bleaching episodes, BMI data from Pulau Weh were compared with data from the GBR in 1998. The BMI of coral genera from the GBR during the 1998 bleaching event were calculated from data taken from Table 2 in Marshall and Baird [11].

### Short-term thermal history of study sites

Historical records of the temperature regime used to develop climatologies and estimate thermal stress at each location were obtained using remotely sensed SST. These were measured by Advanced Very High Resolution Radiometer from the satellite platforms of the US National Oceanic and Atmospheric Administration. Weekly mean SSTs were obtained from the Pathfinder dataset [38] for the centroid of the pixel closest to each study site for a 4 km^2^ grid from 1985 [39] until 2008 and a 50 km^2^ grid from 2009 to 2010 [40]. Data from 1985 to 1996 were used to establish SST climatologies and the MMM temperatures for each location. DHW above MMM for each location between March and August in 1998 and 2010 were estimated by summing thermal anomalies (i.e. weeks where temperatures (°C) were greater than MMM) for the preceding twelve week period. Maximum DHW above MMM was the highest value that occurred during the bleaching event. The method used here differs from the most commonly used DHW method [22] where thermal anomalies only begin to accumulate at temperatures ≥1°C above MMM. Using that approach DHW values ≥4°C-weeks typically result in significant bleaching and DHW values ≥8°C-weeks result in widespread bleaching and significant mortality [40]. We found this approach to severely under-estimate thermal stress at our locations, for example, yielding maximum DHW of only 2.7°C-weeks in Pulau Weh in 2010 despite severe bleaching and mortality (Figures 1 & 2) (Table 2).

### Long-term thermal history of study sites

Long-term thermal histories for each location were determined by examining monthly mean SST data for the 1° longitude–latitude squares that encompassed each study location (5°–6°N & 95°–96°E for Pulau Weh; 1°–2°N and 103°–104°E for Singapore; and 2°–3°N & 104°–105°E for Tioman Island). Data were obtained from HadISST1.1 (Met Office Hadley Centre for Climate Change Global Ocean Surface Temperature dataset; http://badc.nerc.ac.uk/data/hadisst/) for 1951 to 2010 [41]_ENREF_26. SST variability was estimated from the standard deviation of the mean of all monthly mean SST values from January 1951 to December 2010. The long term SST trend (decadal warming rate) was estimated from the regression equation following removal of seasonality using seasonal decomposition (Minitab v. 16).
